# Mechanisms of cisplatin-induced cell death in malignant mesothelioma cells: Role of inhibitor of apoptosis proteins (IAPs) and caspases

**DOI:** 10.3892/ijo.2012.1715

**Published:** 2012-11-28

**Authors:** INEZ L. CREGAN, ARUN M. DHARMARAJAN, SIMON A. FOX

**Affiliations:** 1 School of Anatomy and Human Biology, The University of Western Australia, Crawley, WA;; 2 Molecular Pharmacology Laboratory, School of Pharmacy, Western Australian Biomedical Research Institute & Curtin Health Innovation Research Institute, Curtin University, Bentley, WA, Australia

**Keywords:** mesothelioma IAP, apoptosis, cisplatin, caspase-independent

## Abstract

Malignant mesothelioma (MM) is an aggressive and highly chemoresistant tumour. Although cisplatin is used in frontline therapy of this disease treatment remains palliative at best. The biochemical pathways activated by cisplatin and the mechanisms of resistance in mesothelioma cells are poorly understood. Overexpression of inhibitor of apoptosis proteins (IAPs) has been described in clinical mesothelioma tumours and proposed as therapeutic targets. In this study, we examined cisplatin-induced cell death pathways and IAPs in three mesothelioma-derived cell lines. Cisplatin induced cell death in mesothelioma cell lines was characterised by biochemical mechanisms classically associated with apoptosis including: mitochondrial depolarisation, phosphatidylserine translocation and caspase activation. Surprisingly mRNA expression of IAPs in mesothelioma was not upregulated relative to primary mesothelial cells except for survivin which was higher in the most resistant cell line. In contrast, protein expression of both XIAP and survivin was upregulated in all mesothelioma cells, consistent with post-translational regulation. Knockdown of either XIAP or survivin by RNAi did not affect the sensitivity to cisplatin in any of the cell lines. Survivin RNAi did, however, inhibit proliferation in the highest expressing cell line, ONE58. The pan-caspase inhibitor z-VAD and the more selective caspase 3/7 inhibitor z-DEVD had no effect upon the sensitivity of any of the cell lines to cisplatin indicating that caspase-independent pathways predominate. The findings of the present study provide insights into cisplatin-induced mechanisms in mesothelioma cells and show that alternative pathways are operating which may provide new options for targeting this extremely resistant tumour.

## Introduction

Malignant mesothelioma (MM) is a rare and aggressive tumour, accounting for less than 1% of all cancer deaths in the world (1). Although malignant mesothelioma is rare, it is a particularly aggressive cancer and despite the exploration of a variety of new therapeutic approaches, treatment remains palliative at best (2).

The platinum based compound cisplatin is a widely used chemotherapeutic agent which acts primarily via DNA damage and subsequent induction of apoptotic programs (3). Cisplatin based chemotherapy, either as a single agent or in combination, is commonly employed in therapy of malignant mesothelioma, although its efficacy is limited (2). The antineoplastic activity of cisplatin is rather complex and in light of the published reports it appears that cisplatin may activate several parallel pathways leading to cell cycle arrest and apoptosis depending on the treatment conditions, cell type or concentration (4). Due to this complexity and lack of cytotoxic efficiency in mesothelioma, our knowledge about particular molecular targets of cisplatin in this type of malignancy remains limited despite this compound being widely used in its treatment. Recent evidence from clinical samples has suggested that as for other malignancies inhibitor of apoptosis proteins (IAPs) are upregulated in mesothelioma and may play a role the resistance of these cells to therapy (5–7).

Previous research conducted in our laboratory has characterised the mechanisms of cisplatin-induced apoptosis in malignant mesothelioma cell lines derived from mice (8). The initial aim of this study was to characterise the *in vitro* culture models of human MM, for mechanisms involved in the action of cisplatin (9). Thus in our study we wanted to explore in detail the possible proapoptotic mechanisms of cisplatin in highly aggressive mesothelioma cell lines while also examining the role of IAPs in resistance of this tumour. Various therapies are under development targeting IAPs and in particular XIAP and survivin in a number of malignancies (10) and we wanted to investigate in detail the potential of such therapies in mesothelioma using a panel of cell lines. We found that in these cell lines IAPs were not a factor in cisplatin resistance and that cisplatin instead targets caspase-independent pathways.

## Materials and methods

### Cell culture and reagents

The malignant mesothelioma cell lines JU77, LO68 and ONE58 were used in this study. These cell lines were originally derived from pleural effusions of different patients presenting with malignant pleural mesothelioma (9). All cells were cultured and maintained in medium R5, which is RPMI-1640 plus 5% heat-inactivated fetal bovine serum (FBS), 300 mM L-glutamine, 120 *μ*g/ml penicillin and 100 *μ*g/ml gentamicin (all supplied by Invitrogen, Victoria, Australia). All cell cultures were grown at 37°C in a 5% CO_2_ humidified atmosphere. Mesothelial cell RNA and protein was kindly provided by Dr Steve Mutsaers (Lung Institute of Western Australia).

### Cell viability

An MTT assay was used to quantitate cell death/viability following 24 h exposure to cisplatin. Cells were seeded into 96-well plates at a density of 20,000 cells/well. Following 24 h incubation, cisplatin was added at concentrations from 0–100 *μ*g/ml and the cells incubated for a further 24 h. The assay was terminated by aspirating the media, 100 *μ*l of 1 mg/ml MTT (Sigma-Aldrich, NSW, Australia) in RPMI was added and the plates incubated for 60 min at 37°C. Following removal of the supernatant 100 *μ*l of DMSO was added to solubilise the dye and absorbance measured at 595 nm with a microplate reader (Model 3550, Bio-Rad Laboratories, CA, USA). IC_50_ was defined as the concentration causing a 50% reduction in absorbance relative to the negative control. IC_50_ was determined by non-linear regression analysis using Graphpad Prism v4 (Graphpad Software, CA, USA).

### Annexin-V and propidium iodide staining

Annexin-V and Propidium Iodide (PI) staining was performed using an Annexin-V-Fluos kit (Roche Diagnostics, NSW, Australia). Cells were seeded into 6-well plates at a density of 5×10^5^ cells/well. Following 24 h incubation cisplatin was added at concentration 0–100 *μ*g/ml and the cells incubated for a further 24 h. Cells were harvested by trypsinization, collected by centrifugation, washed once with 1 ml of PBS and stained according to the manufacturers’ protocol and analysed using a FACS Calibur Flow Cytometer (BD Biosciences, NSW, Australia).

### Determination of the mitochondrial transmembrane potential

ΔΨ_m_ was analysed using the cationic dye JC-1 (5, 5′, 6, 6′-tetrachloro-1, 1′, 3, 3′-tetraethyl-benzimidazolcarbocyanine iodide) which exhibits potential-dependent accumulation in mitochondria by fluorescence emission shift from green (∼525 nm) to red (∼590 nm). Consequently, mitochondrial depolarization is indicated by a decrease in the red/green fluorescence intensity ratio. Cells were seeded into black 96-well plates (Greiner, Germany) at a density of 10,000 cells/well in 100 *μ*l R5. Following 24 h incubation, cisplatin was added at concentrations from 0–100 *μ*g/ml and the cells were incubated for a further 24 h. The media was then aspirated and 50 *μ*l of staining solution added [33 *μ*M JC-1 (Invitrogen) in serum free R-5], and the plates incubated for 1 h at 37°C, 5% CO_2_. The staining solution was then removed and 200 *μ*l of PBS with 5% BSA was added. After a further 5-min incubation, PBS/BSA was removed and 100 *μ*l PBS was added. The plates were read on a Fluostar Optima plate reader (excitation filter 485 nm and emission filters 520 nm /595 nm) (BMG Laboratories, Victoria, Australia). Data are presented as the ratio of red to green. FCCP [carbonyl cyanide 4-(trifluoromethoxy)phenylhydrazone] (Sigma-Aldrich) was used as a positive control for maximal mitochondrial depolarisation.

### Caspase activity assay

Activation of effector caspases during apoptosis was determined using the Caspase-3 Assay kit#2 (Invitrogen) with a protocol modified for a direct homogeneous assay. Cells were seeded into black 96-well plates (Greiner) at a density of 20,000 cells/well in 100 *μ*l medium. Following 24 h incubation, cisplatin was added at concentrations from 0–100 *μ*g/ml and the cells incubated for a further 24 h. The assay was terminated by the addition of 100 *μ*l of reagent buffer (5 *μ*l 20X cell lysis buffer, 20 *μ*l 5X reaction buffer, 0.5 *μ*l substrate z-DEVD-R110, 0.5 *μ*l of 1M DTT, 74 *μ*l dH_2_O) and the fluorescent signal measured after 1 h (Fluostar Optima, BMG Laboratories). Appropriate blank and control samples were included in each run as recommended by the assay manufacturer. Caspase inhibitors were the pan-caspase inhibitor z-VAD-fmk and the caspase 3/7 inhibitor z-DEVD-cmk (Calbiochem, Victoria, Australia).

### Reverse transcription PCR (RT-PCR) and real-time RT-PCR

Total RNA was isolated from cultures of the cell lines using Ultraspec RNA reagent (Biotec, TX, USA) according to the manufacturer’s instructions. The RNA pellet was resuspended in 1 mM sodium citrate buffer and samples were stored at −80°C. Prior to RT-PCR, contaminating DNA was removed from the RNA using RQ1 DNAse (Promega, NSW, Australia). First strand cDNA synthesis was performed using SuperScript III First-Strand Synthesis System with oligo dT primers (Invitrogen). Gene specific PCR primers were designed using the Primer 3 software (11) and the sequences are shown in Table I. Real-time PCR was performed using a RotorGene real-time amplification instrument (Corbett Research, NSW, Australia) using a SensiMix SYBR kit (Bioline, NSW, Australia) according to the manufacturers’ instructions. A melt curve analysis was performed at end of each experiment to confirm specificity.

Gene expression data were normalised to levels of expression of reference (housekeeping) genes GAPDH and HPRT1. PCR primers were as follows: survivin (NM_001168) forward (5′-cttgaaagtggcaccagagg-3′), reverse (5′-ggaccaccgcatct ctacat-3′); XIAP (NM_001167) forward (5′-ggggttcagtttcaa gga-3′), reverse (5′-cgccttagctgctcttcagt-3′); IAP1 (NM_001165) forward (5′-cctggatagtctactaactgcct-3′), reverse (5′-gcttct tcagagagtttctgaa-3′); IAP2 (NM_001160) forward (5′-cagaat tggcaagagctggt-3′), reverse (5′-attcgagctgcatgtgtct-3′); HPRT1 (NM_000194) forward (5′-tgacactggcaaaacaatgca-3′), reverse (5′-ggtccttttcaccagcaagct-3′) and GAPDH (NM_002046) forward (5′-accacagtccatgccatcac-3′), reverse (5′-tccaccacc ctgttgctgta-3′). Real-time assays were performed on the relevant samples and from the reference gene data generated for each sample using geNorm software (v3.3) essentially as described by Vandesompele *et al*(12). Relative expression of the target gene was normalised using this factor and expressed as mean ± standard deviation relative to a control or calibrator sample.

### Immunoblot analysis

Cell monolayers were washed once with ice-cold saline and lysed by addition of RiPa buffer [50 mM Tris-HCl (pH 8.0), 150 mM NaCl, 1% Triton X-100, 0.5% Na deoxycholate, 0.1% SDS) with protease inhibitors (Roche Biochemicals)]. Incubated for 1 h at 4°C with gentle agitation then harvested with a cell scraper and transferred to a 1.5 ml tube. The lysate was then centrifuged at 12,000 g for 30 min. at 4°C and the supernatants recovered and stored at −80°C. Protein concentrations were determined by DC protein assay (Bio-Rad). Proteins were resolved on 4–12% NuPAGE polyacrylamide gels (Invitrogen) and transferred to nitrocellulose membrane (Pall, Victoria, Australia) and probed with the following antibodies: anti-XIAP (Cell Signaling Technology, MA, USA), anti-survivin (BioLegend, CA, USA) and anti-actin (Sigma-Aldrich). After incubation with alkaline phosphatase conjugated secondary antibody, colour was developed using 5-bromo-4-chloro-3-indolyl phosphate/nitroblue tetrazolium reagents (Sigma-Aldrich).

### Cell-based ELISA

A cell-based ELISA assay was used to monitor changes in protein expression in response to RNAi duplex transfection. Preliminary experiments were performed to optimise the conditions for each cell line with respect to cell density and antibody concentration. Following treatment in 96-well plates, the experiment was terminated by removing the media and fixing the cells with ice-cold methanol for 10 min. The methanol was aspirated and the cells were rinsed 3 times for 5 min each with 200 *μ*l per well of 0.1% Triton X-100 in PBS. The cells were incubated for 5 min with 100 *μ*l of 3% H_2_O_2_ (to block endogenous peroxidase activity), washed twice with 0.5% Tween-20 in PBS (wash buffer) and then blocked with 5% FBS in PBS for 30 min. Cells were washed 3 times, incubated overnight with primary antibody (as for immunoblot) and following three washes incubated at room temperature for 1 h with HRP conjugated secondary antibody. Detection was performed for 1 h with ABTS (2,2′-azinodiethyl-benzthiazoline sulfonate) substrate (Sigma-Aldrich), stopped with 1% SDS and absorbance measured at 405 nm. Appropriate background and antibody controls were included in each assay.

### RNAi-mediated gene silencing

The siRNA duplexes were Stealth Select 3 RNAi Sets for human XIAP and survivin (Invitrogen) and the optimal duplex as determined empirically for each cell line was used for the experiments presented here. The negative control duplexes were the appropriate (based upon GC content) Stealth siRNA negative controls (Invitrogen). Transfection of RNAi duplexes was performed using Lipofectamine (Invitrogen, Australia) essentially according to the manufacturers recommendation with concentration determined empirically.

## Results

### Mechanisms of cisplatin induced cell death in mesothelioma cells

**Figure 1.**
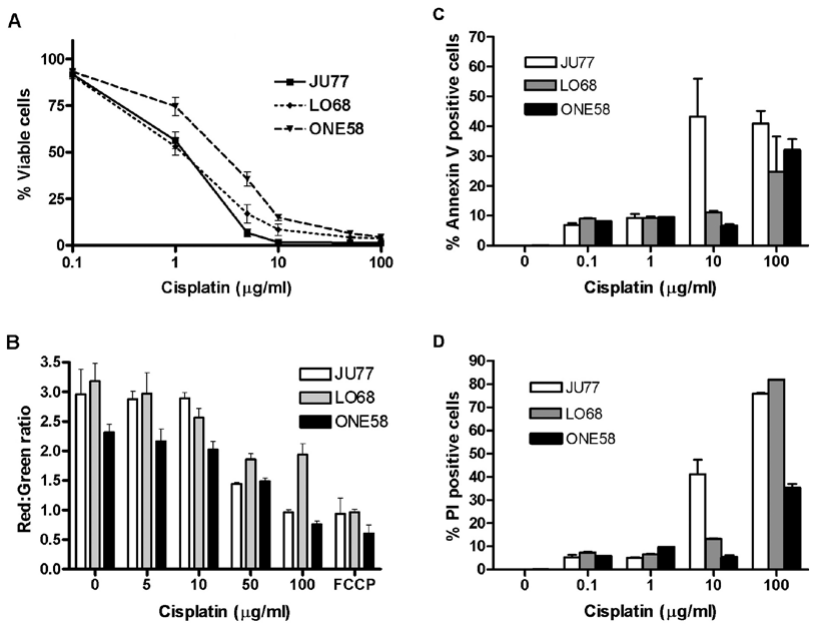
Mechanisms of cisplatin induced cell death in mesothelioma cells. (A) *In vitro* sensitivity of MM cell lines to cisplatin. Cells were cultured in the presence of cisplatin for 24 h. Cell viability was determined by MTT assay. Data presented are the mean ± SD of five independent experiments each performed in triplicate. (B) Dose-dependent mitochondrial membrane depolarisation. Mitochondrial membrane potential was measured by JC-1 accumulation following cisplatin treatment (lower ratio of red/green indicates loss of potential). Results are the mean ± SD of at least three independent treatments performed in triplicate. FCCP-positive control. (C) Phosphatidylserine (PS) translocation in response to cisplatin. PS translocation to the outer cell membrane was measured by Annexin-V binding and flow cytometry. Cells were treated with a range of cisplatin concentrations (0.1–100 *μ*g/ml) for 24 h. Results shown are the mean ± SD of two independent experiments performed in triplicate. (D) Loss of membrane integrity in response to cisplatin. Membrane integrity was measured by staining with the cell impermeant dye propidium iodide and flow cytometry. Results shown are the mean ± SD of two independent experiments performed in triplicate.

In order to examine the mechanisms of cisplatin effect upon mesothelioma we initially evaluated the *in vitro* sensitivity of three mesothelioma cell lines, LO68, ONE58 and JU77 to 24 h exposure to cisplatin (0.1–100 *μ*g/ml) by MTT assay. Results were determined by constructing dose-response curves (Fig. 1A) and the IC_50_ calculated by further non-linear regression analysis of the data. The results showed that JU77 (IC_50_ of 1.14±0.21 *μ*g/ml) and LO68 (IC_50_ of 1.2±0.45 *μ*g/ml) were the most cisplatin sensitive cell lines when compared to ONE58 (IC_50_ of 3.08±0.83 *μ*g/ml).

Given the importance of mitochondria in cell death regulation and apoptotic signalling we examined mitochondrial functional integrity in cisplatin treated mesothelioma cells. Mitochondrial disruption and the loss of mitochondrial membrane potential (MMP) was assayed using the fluorescent mitochondrial potential sensor (JC-1). We found that all three mesothelioma cell lines exhibited dose-dependent loss of MMP following cisplatin treatment (Fig. 1B). Interestingly, at 100 *μ*g/ml in both JU77 and ONE58 cisplatin inhibited the function of mitochondria to the same extent as the positive control FCCP (a protonophore that dissipates the H+ gradient across the inner membrane of mitochondria) whereas a lesser effect was seen in LO68 cells.

In order to further explore the specific mechanisms associated with cisplatin-induced cell death in this tumour cell type, the biochemical changes in the cell membrane associated with apoptosis were measured (Fig. 1C and D). All three cell lines demonstrated increased Annexin-V binding indicative of phosphatidylserine translocation in response to cisplatin treatment, which was most pronounced in JU77 cells (Fig. 1C). When PI staining showing loss of membrane integrity was determined, JU77 cells also showed response at a lower dose than LO68 or ONE58 cells (Fig. 1D). These results were consistent with cell viability data showing JU77 as the most sensitive (Fig. 1A).

### Caspase activation and IAP expression in MM cells

**Figure 2.**
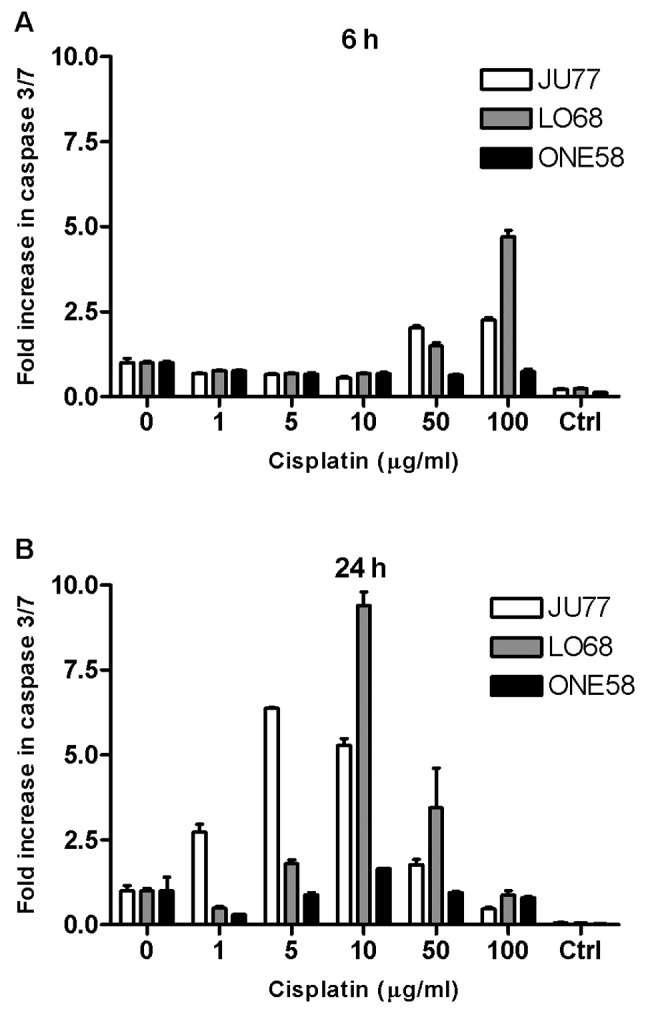
Dose- and time-dependent caspase activation by cisplatin. Caspase activity was measured (A) 6 h and (B) 24 h following cisplatin treatment. Data are presented as fold increase in fluorescence as measured relative to untreated cultures. Data are the mean ± SD of two independent treatments performed in triplicate. Ctrl cells treated with caspase 3/7 inhibitor ac-DEVD-cho.

We char-acterised time- and dose-dependent patterns of cisplatin induced caspase-3/7 activation in these cell lines. Cultures of JU77, LO68 and ONE58 were treated with cisplatin and dose-related changes in caspase 3/7 activity were assayed at 0, 3, 6 and 24 h. All MM cell lines demonstrated an increase in caspase 3/7 activation in response to cisplatin (Fig. 2), although no effect was seen at 3 h (not shown). Caspase 3/7 activation occurs at a later time with lower doses of cisplatin (Fig. 2B), whereas with higher doses of cisplatin, caspase 3/7 activation occurs earlier (Fig. 2A). In JU77 increases in caspase 3/7 activity was demonstrated at low concentrations of cisplatin (1–10 *μ*g/ml) after 24 h of exposure (Fig. 2A). Interestingly, the most pronounced upregulation in caspase activity was seen in LO68 cells. In contrast, overall caspase 3/7 activation in ONE58 cells was only seen at 10 *μ*g/ml at 24 h and in no other sample.

**Figure 3.**
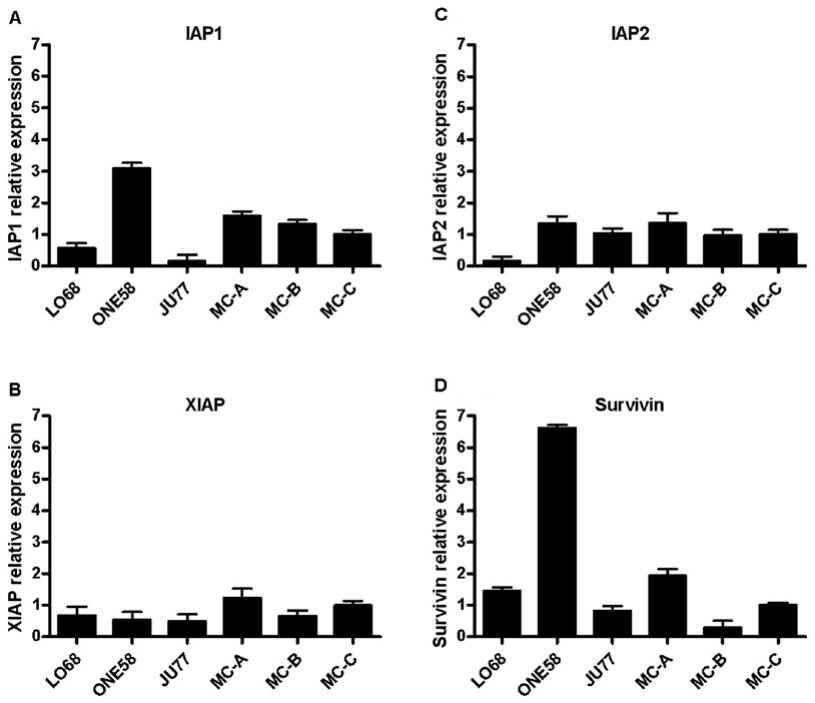
Basal gene expression of (A) IAP1 (B) XIAP (C) IAP2 and (D) survivin in mesothelioma and primary mesothelial cells (MC). Total RNA isolated from cells was analysed by 2-step real-time RT-PCR using gene specific primers. Basal gene expression is expressed as mRNA levels relative to mesothelial culture C (MC-C) following normalisation by reference gene expression. Results are means ± SD for three cultures.

**Figure 4.**
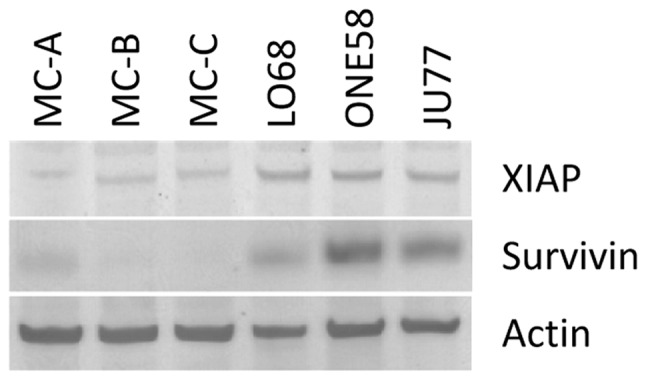
IAPs are differentially expressed at the protein level in mesothelioma cells. Proteins extracted from mesothelioma and three different primary mesothelial cell cultures (MC) were analysed by immunoblotting with relevant antibodies. Results are representative of three different determinations.

We next investigated the expression of IAPs in these cells since reports have indicated overexpression of some of these molecules in MM (5,6) with implications for apoptosis resistance. Using conventional PCR we found that IAP-1, IAP-2, XIAP, and survivin mRNAs were expressed in all MM cell lines (data not shown) and we went on to examine differential expression of these genes in MM cells and primary mesothelial cell cultures (Fig. 3). We found that gene expression of both IAP1 and survivin were upregulated in ONE58 cells (Fig. 3A and D) which may account for the lack of caspase activation in these cells but that otherwise there was no clear pattern of expression difference between the MM cell lines and mesothelial cultures. Since some authors (13,14) have reported that cisplatin can downregulate IAP expression in tumour cells we also examined the mRNA levels of IAPs by real-time PCR following 3, 6 and 24 h of treatment with 10 *μ*g/ml cisplatin but we did not see any significant changes in IAP expression in MM cells (data not shown). To further investigate IAP expression in these cells we performed immunoblotting studies of XIAP and survivin protein expression (Fig. 4) which demonstrated higher levels of both these proteins in the MM tumour cell lines than in primary cells. Given the results of the mRNA analysis of these genes (Fig. 3B and D) this suggests a level of translational regulation of these molecules.

### Silencing of XIAP and survivin in mesothelioma cells

Since immunoblotting studies indicated differential expression of both XIAP and survivin protein in mesothelioma cell lines (Fig. 4) we further investigated the role of these proteins in resistance to cisplatin using RNAi mediated knockdown. Previously, in our laboratory (unpublished data) we observed that mRNA knockdown by RNAi may not correlate with protein. Therefore, we employed cell based ELISA assays for XIAP and survivin to optimise for RNAi mediated knockdown at the protein level.

**Figure 5.**
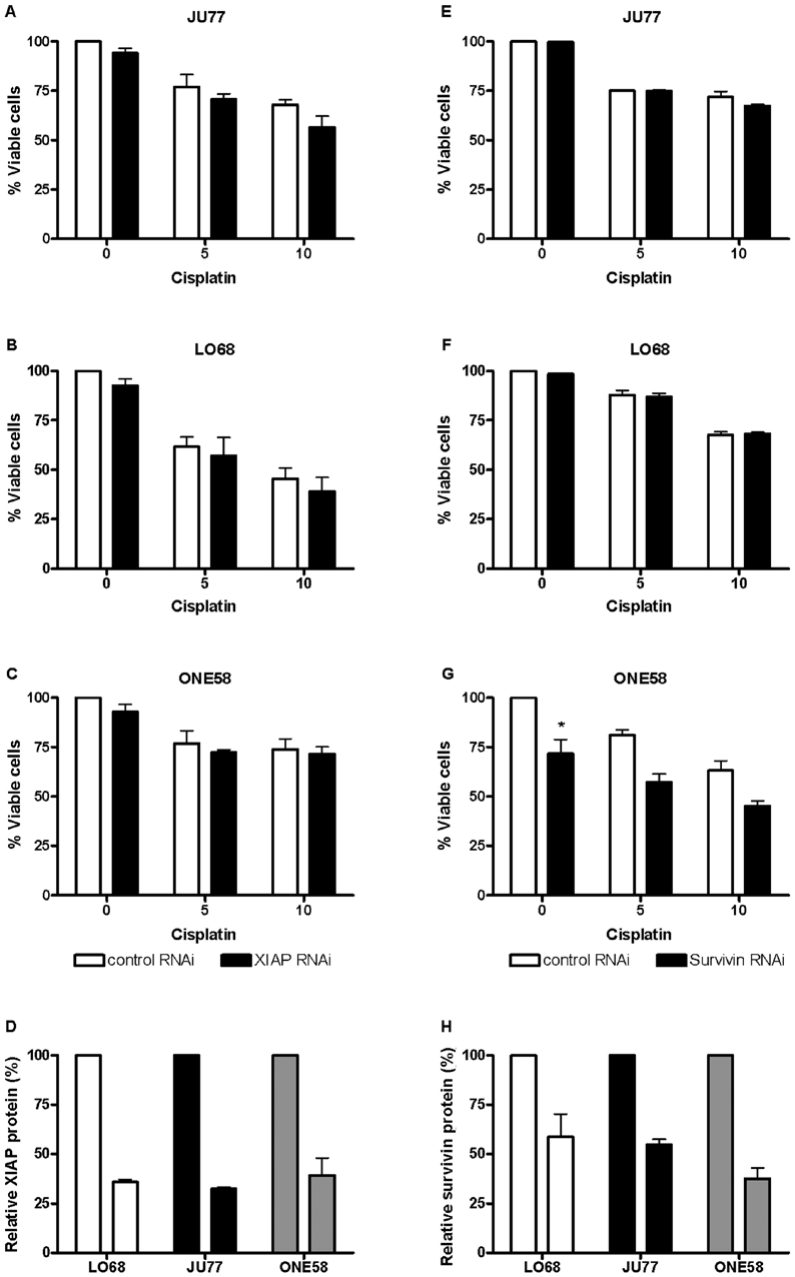
Effect of IAP knockdown by RNAi upon *in vitro* sensitivity to cisplatin. (A) JU77 XIAP (B) LO68 XIAP (C) ONE58 XIAP (E) JU77 survivin (F) LO68 survivin (G) ONE58 survivin. Cells were transfected with optimal RNAi duplexes and then incubated for 48 h for optimal knockdown and cultured in the presence of cisplatin (5 and 10 *μ*g/ml) for 24 h. Cell viability was determined by MTT assay. Each bar represents the mean ± SD from three independent experiments. ^*^There was a significant decrease in cell viability in ONE58 cells following survivin knockdown p<0.05 (Fisher’s t-test). Extent of IAP protein knockdown by RNAi: (D) XIAP (H) survivin. Cells were transfected as above and after 48 h assayed by cell ELISA for relative protein expression. Each bar represents the mean ± SD from two independent experiments.

In all three cell lines we were able to establish conditions which achieved optimal knockdown to at least 40% of XIAP (Fig. 5D) 48–72 h after transfection. Experiments in JU77, LO68 and ONE58 showed that knockdown of XIAP had little effect upon the sensitivity of these cell lines to cisplatin (Fig. 5A–C). Subsequently a similar series of experiments was conducted using RNAi mediated knockdown of survivin. It proved more difficult in these cells to knock down survivin at the protein level (Fig. 5H) than XIAP, although the effect in ONE58 cells (the highest expressing) was comparable. Similarly the sensitivity of JU77 and LO68 cells (Fig. 5E and F) to cisplatin was not affected by survivin knockdown. In ONE58 cells survivin protein downregulation had a significant effect upon baseline cell viability (Fig. 5G) although this was independent of cisplatin treatment.

### Caspase independence of cisplatin-induced cell death in mesothelioma cells

**Figure 6.**
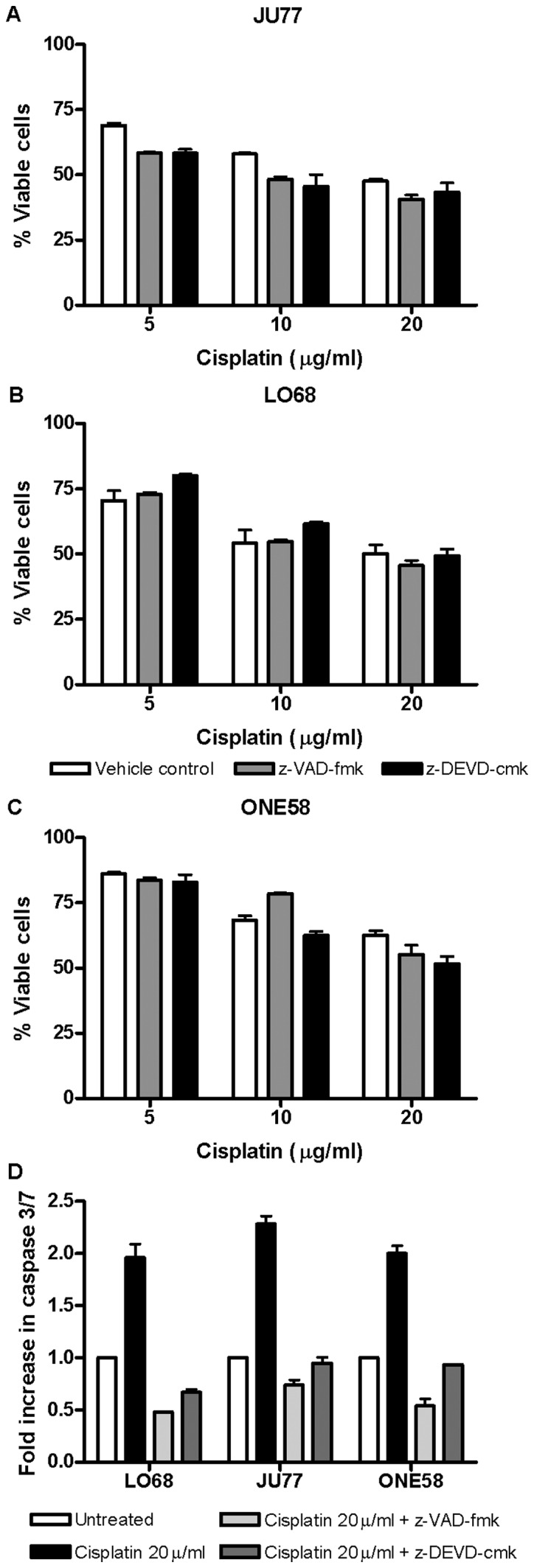
Effect of caspase inhibition upon *in vitro* sensitivity to cisplatin. (A) JU77 (B) LO68 (C) ONE58. Cells were cultured in the presence of cisplatin (0–20 *μ*g/ml) for 24 h following exposure to caspase inhibitors. Cell viability was determined by MTT assay. Results are mean ± SD of three independent treatments performed in triplicate. (D) Caspase inhibitors effectively block cisplatin induced caspase activation. Cells were incubated with a range of caspase inhibitors and exposed to cisplatin (0–20 *μ*g/ml) for 24 h. Caspase activity was measured after 24 h and expressed as fold increase relative to untreated cells. Results are the mean ± SD of at least two independent treatments performed in triplicate.

Given the lack of effect of knockdown of XIAP and survivin upon drug cytotoxicity we hypothesised that caspase activation may be unnecessary for the effect of cisplatin in these cells. Therefore, we investigated the effect of both the pan-caspase inhibitor z-VAD-fmk and the caspase 3/7 inhibitor z-DEVD-cmk upon these cells. Both of these inhibitors effectively blocked caspase 3/7 activation by cisplatin (Fig. 6D). Neither broad nor the more narrow spectrum caspase inhibition significantly influenced cisplatin induced cytotoxicity in mesothelioma cells (Fig. 6A–C). These results suggest that in these cell lines caspase activation is not essential for cell death due to cisplatin and most likely caspase-independent pathways predominate.

## Discussion

In the present study, we have characterised the cisplatin chemosensitivity of malignant mesothelioma cell lines (JU77, LO68 and ONE58), and investigated in detail the mechanisms of cell death induced by this chemotherapeutic which is commonly employed in the clinical treatment of MM. Conventionally the best understood pathway of cisplatin toxicity is activation of DNA damage signalling pathways which trigger mitochondrial apoptosis (15). Our initial experiments established the cytotoxic effect and relative sensitivity of 3 mesothelioma cells lines to cisplatin. Examination of biochemical mechanisms induced by cisplatin demonstrated changes generally associated with apoptotic cell death including mitochondrial depolarisation and characteristic membrane changes. The active involvement of caspases is considered one of the hallmarks of cytotoxic drug-induced apoptosis. We focussed upon the executioner caspase 3/7 and found activation in both JU77 and LO68 but much less in ONE58 (the most resistant cell line).

We next investigated basal expression of IAPs in these cells given the purported role of these molecules in resistance to drug induced cytotoxicity (10). Survivin mRNA was upregulated in ONE58 consistent with the low caspase activation in these cells. However, apart from this we did not see the difference in XIAP and survivin expression that was expected from the literature (5–7). While upregulation of basal IAPs have been reported in numerous studies few have examined changes in IAP gene expression in response to cisplatin treatment. We did not find regulation of IAP gene expression in response to cisplatin treatment in the present study. Results of other studies have provided conflicting evidence for the regulation of IAPs in response to chemotherapeutic drugs including cisplatin (13,14,16). Our results are consistent with this being a cell type- and drug-dependent mechanism which does not operate downstream of cisplatin in mesothelioma cells.

Surprisingly, immunoblotting revealed differential expression of both XIAP and survivin at the protein level which was not seen in mRNA, with the exception of survivin in ONE58 cells. While many studies have focussed upon differences in mRNA levels of these proteins there is also evidence for significant regulation of both at the protein level (10). Both XIAP (17) and survivin (18) have been shown as regulated through mechanisms controlling the proteasome degradation pathway. In addition there is evidence for other posttranslational mechanisms functioning to regulate these proteins (reviewed in refs. 19 and 20).

Our results for protein expression were consistent with the few studies which have investigated IAP molecules in clinical samples of mesothelioma (6,7). However, a role for these proteins in resistance to cisplatin was not borne out by our subsequent experiments. The XIAP protein knockdown achieved by us was at a level consistent with similar studies in other cell types which showed effects upon viability and drug sensitization (21,22). Our data indicate that inhibition of caspase activation by XIAP was not likely to be important in the resistance of mesothelioma cell lines to cisplatin.

The frequent overexpression of survivin in cancer is well recognised and often associated with resistance to therapy poor prognosis and an aggressive progression (10), therapies targeting this molecule are in various stages of development (23). We achieved a 40–60% knockdown of this protein, however, as with XIAP, there was no effect upon cisplatin sensitivity. Most studies have merely assessed cell viability directly following survivin knockdown rather than in conjunction with drug treatment and we did observe a significant effect upon cell viability in ONE58 cells. Targeting survivin by RNAi in other cell types combined with drugs has shown conflicting results with sensitization (24) or no effect (25). Several previous studies have examined the functional effects of inhibition of survivin in mesothelioma cell lines finding enhanced caspase activation (7,26) and apoptotic morphology (7) in response to cisplatin. In the present study we used a panel of three distinct mesothelioma cell lines and assessed viability along with other apoptotic mechanisms, caspase activation was observed (unpublished data) but this did not affect cell viability.

In addition to more recently described mechanisms, inhibition of caspase activation either directly by XIAP or indirectly by survivin remains an important function of these proteins (10). We speculated that the absence of effect by RNAi in our experiments may relate to caspase activation. Our finding that cisplatin induced cell death in mesothelioma cells did not require caspase activation confirmed this. To our knowledge this is the first time that caspase-independent pathways have been identified as prominent in mesothelioma cells response to chemotherapy.

Classical apoptosis pathways involving caspase activation are the most widely described cisplatin induced mechanisms (15) although there is a growing body of evidence for caspase independent pathways (27–30). These pathways are increasingly of interest because they represent a level of redundancy in cell death signalling which might be therapeutically exploited in tumours which are inherently resistant to apoptosis (31). Notable in the present study was the commonality of the findings regarding caspase independence in all three cell lines under investigation. Current studies in our laboratory are directed at elucidating the alternate pathways operating in these cells.

## Figures and Tables

**Table I.** The sequences used.

Gene	Primer sequence 5′-3′	Genbank accn.
Survivin	CTTGAAAGTGGCACCAGAGG	NM_001168
	GGACCACCGCATCTCTACAT	
Bruce	TCAACAGGTGAGTGCTCCAG	NM_016252
	TCCAAAAGCAGCCAAAGAAT	
Livin	CTGGTCAGAGCCAGTGTTCC	NM_139317
	TCATAGAAGGAGGCCAGACG	
XIAP	GGGGTTCAGTTTCAAGGA	NM_001167
	CGCCTTAGCTGCTCTTCAGT	
IAP1	CCTGGATAGTCTACTAACTGCCT	NM_001166
	GCTTCTTGCAGAGAGTTTCTGAA	
IAP2	CAGAATTGGCAAGAGCTGGT	NM_001165
	ATTCGAGCTGCATGTGTCT	

## References

[1] Spugnini EP, Bosari S, Citro G, Lorenzon I, Cognetti F, Baldi A (2006). Human malignant mesothelioma: molecular mechanisms of pathogenesis and progression. Int J Biochem Cell Biol.

[2] Ray M, Kindler HL (2009). Malignant pleural mesothelioma: an update on biomarkers and treatment. Chest.

[3] Rabik CA, Dolan ME (2007). Molecular mechanisms of resistance and toxicity associated with platinating agents. Cancer Treat Rev.

[4] Koberle B, Tomicic MT, Usanova S, Kaina B (2010). Cisplatin resistance: preclinical findings and clinical implications. Biochim Biophys Acta.

[5] Davidson B (2007). Expression of cancer-associated molecules in malignant mesothelioma. Biomark Insights.

[6] Kleinberg L, Lie AK, Florenes VA, Nesland JM, Davidson B (2007). Expression of inhibitor-of-apoptosis protein family members in malignant mesothelioma. Hum Pathol.

[7] Zaffaroni N, Costa A, Pennati M (2007). Survivin is highly expressed and promotes cell survival in malignant peritoneal mesothelioma. Cell Oncol.

[8] Fox SA, Kusmiaty, Loh SS, Dharmarajan AM, Garlepp MJ (2005). Cisplatin and TNF-alpha downregulate transcription of Bcl-xL in murine malignant mesothelioma cells. Biochem Biophys Res Commun.

[9] Manning LS, Whitaker D, Murch AR (1991). Establishment and characterization of five human malignant mesothelioma cell lines derived from pleural effusions. Int J Cancer.

[10] Altieri DC (2010). Survivin and IAP proteins in cell-death mechanisms. Biochem J.

[11] Rozen S, Skaletsky H (2000). Primer3 on the WWW for general users and for biologist programmers. Methods Mol Biol.

[12] Vandesompele J, De Preter K, Pattyn F (2002). Accurate normalization of real-time quantitative RT-PCR data by geometric averaging of multiple internal control genes. Genome Biol.

[13] Matsumiya T, Imaizumi T, Yoshida H, Kimura H, Satoh K (2001). Cisplatin inhibits the expression of X-chromosome-linked inhibitor of apoptosis protein in an oral carcinoma cell line. Oral Oncol.

[14] Nomura T, Mimata H, Yamasaki M, Nomura Y (2004). Cisplatin inhibits the expression of X-linked inhibitor of apoptosis protein in human LNCaP cells. Urol Oncol.

[15] Siddik ZH (2003). Cisplatin: mode of cytotoxic action and molecular basis of resistance. Oncogene.

[16] Andjilani M, Droz JP, Benahmed M, Tabone E (2006). Down-regulation of FAK and IAPs by laminin during cisplatin-induced apoptosis in testicular germ cell tumors. Int J Oncol.

[17] Golovine K, Makhov P, Uzzo RG (2010). Cadmium down-regulates expression of XIAP at the post-transcriptional level in prostate cancer cells through an NF-kappaB-independent, proteasome-mediated mechanism. Mol Cancer.

[18] Zhao J, Tenev T, Martins LM, Downward J, Lemoine NR (2000). The ubiquitin-proteasome pathway regulates survivin degradation in a cell cycle-dependent manner. J Cell Sci.

[19] Altieri DC (2008). New wirings in the survivin networks. Oncogene.

[20] Holcik M (2003). Translational upregulation of the X-linked inhibitor of apoptosis. Ann N Y Acad Sci.

[21] Li Y, Jian Z, Xia K (2006). XIAP is related to the chemoresistance and inhibited its expression by RNA interference sensitize pancreatic carcinoma cells to chemotherapeutics. Pancreas.

[22] Zhang Y, Wang Y, Gao W (2006). Transfer of siRNA against XIAP induces apoptosis and reduces tumor cells growth potential in human breast cancer in vitro and in vivo. Breast Cancer Res Treat.

[23] Mita AC, Mita MM, Nawrocki ST, Giles FJ (2008). Survivin: key regulator of mitosis and apoptosis and novel target for cancer therapeutics. Clin Cancer Res.

[24] Liu WS, Yan HJ, Qin RY (2009). siRNA directed against survivin enhances pancreatic cancer cell gemcitabine chemosensitivity. Dig Dis Sci.

[25] Yamaguchi Y, Shiraki K, Fuke H (2005). Targeting of X-linked inhibitor of apoptosis protein or survivin by short interfering RNAs sensitize hepatoma cells to TNF-related apoptosis-inducing ligand- and chemotherapeutic agent-induced cell death. Oncol Rep.

[26] Hopkins-Donaldson S, Belyanskaya LL, Simoes-Wust AP (2006). p53-induced apoptosis occurs in the absence of p14(ARF) in malignant pleural mesothelioma. Neoplasia.

[27] Cummings BS, Kinsey GR, Bolchoz LJ, Schnellmann RG (2004). Identification of caspase-independent apoptosis in epithelial and cancer cells. J Pharmacol Exp Ther.

[28] Kim R, Emi M, Tanabe K (2005). Caspase-dependent and -independent cell death pathways after DNA damage (Review). Oncol Rep.

[29] Liu L, Xing D, Chen WR (2009). Micro-calpain regulates caspase-dependent and apoptosis inducing factor-mediated caspase-independent apoptotic pathways in cisplatin-induced apoptosis. Int J Cancer.

[30] Lock EA, Reed CJ, Kinsey GR, Schnellmann RG (2007). Caspase-dependent and -independent induction of phosphatidylserine externalization during apoptosis in human renal carcinoma Cak(1)-1 and A-498 cells. Toxicology.

[31] Delavallee L, Cabon L, Galan-Malo P, Lorenzo HK, Susin SA (2011). AIF-mediated caspase-independent necroptosis: a new chance for targeted therapeutics. IUBMB Life.

